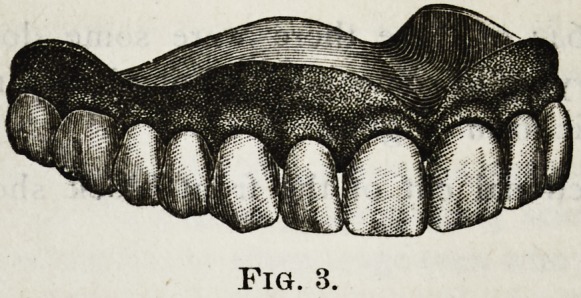# To Whom It May Concern

**Published:** 1889-12

**Authors:** 


ARTICLE VII.

### TO WHOM IT MAY CONCERN.

### PATENTS CONSIDERED IN RELATION TO THE PATENTEES AND THE PUBLIC.

ARTICLE 1, section 8 of the Constitution of the United States says:

"Congress shall have power to promote the progress of science and all the useful arts by securing for limited times to authors and inventors the exclusive right to their respective writings and discoveries."

The Patent Laws of 1790, 1793, 1836, 1861 and others are entitled "Act to promote the useful arts."

Laws conferring upon individuals or corporations special privileges or immunities are made upon the theory that the general public, which is represented by the law-makers, is to be benefited. Substantially the people say, "For a prospective gain to all, we will give you certain limited monopolies and relieve you of some obligations to encourage you to employ your time in ways, and to undertake enterprises, which will be for the good of the public."

The term monopolies is not used here in any offensive sense, but in its literal meaning.

The individual asking for a patent which shall protect him in the sole use of his invention is not supposed to be acting in the interest of the public, but for his own financial advantage; neither does the law give him a patent because it helps the claimant to get rich, but, as we have seen, for other and public reasons.

With this view of the purpose of the laws for conferring patents, it ought not to be difficult to determine the conditions upon which they should be granted. First, in the language of the Constitution, "to promote the progress of science and all the useful arts."

This title is broad enough to cover the whole ground, and it is reiterated in some form in the title of every act creating or amending the Patent Laws.

The plain language of the Patent Laws of the United States is that the claimant should be the first and original inventor of the thing claimed, also that it should be the result of "effort, industry or genius" in the claimant. When these conditions are complied with no one can reasonably object to the granting of a patent, and the purpose of the law, "to promote the progress of science and all the useful arts," will be attained.

Keeping the distinction plainly in view between the purpose of the people in granting patents, and that of the inventor in soliciting the grant, we shall see that certain considerations ought not to influence the decisions of the Patent Office.

First, the fact that the patents granted by the Commissioner may be revised by the courts should not be allowed to influence his decisions.

Often, apparently, this is not the case. Too often it seems plain that well-paid solicitors have succeeded in worrying the officials into favorable reports on the most trivial applications for inventions either worthless, or requiring a great stretch of the imagination to find anything new, or any evidence of "effort, industry or genius" in them.

Such decisions in the Patent office afford unscrupulous men an opportunity to hinder the business of other manufacturers, or extort royalties to which they have no moral right, although they may legally claim them.

Such patents act directly against the purpose for which the laws were made; instead of developing science and art, the tendency of such is to discourage men who are working to develop something useful and practical; they feel that there is no security for their labor, and that until they have been through a long and expensive suit or series of suits at law they will not know whether the work of their brains is to be legally their own or that of someone who has, by the aid of a long purse, succeeded against right and justice.

As an example of the class of patents that ought not to be granted, take one that has been recently issued for the form of the gum of artificial teeth. The claim is for the corrugated appearance and marginal swell near the border of the gum.

This patent, it will be observed, is for the form of the artificial gum as connected with artificial teeth.

Now the parties who obtained this patent claim to make the most perfect imitations of the natural teeth and gums, and therefore the claim in their patent is for something which is only a copy of a well-known natural form, and not an invention, the result of an expenditure of "effort industry, or genius," and therefore, under the law, clearly not patentable.

Again, suppose the form were patentable, the law requires that the claimant shall be the first and original inventor, to entitle him to a patent.

In this case the patentees were not the first to copy this form, as shown by the cuts below.

These cuts are from samples taken from the stock of Mr. H. D. Justi, of Philadelphia, and clearly show that long before this patent he had not overlooked this peculiarity in the form of the gum, but had accurately modeled it in his own factory.

Cut No. 1. shows a form of teeth and gum made in the year 1864; of these there were some dozen different moulds, varying in some points, but all having the same characteristic form of gum.

The sectional cut of the front block shows the corrugation.

FIG. 1.

Cut No. 2 represents a complete set of teeth which, with others of the same form, was exhibited at the Centennial Exposition in Philadelphia, 1876.

It will be seen that the corrugated gum and the swell near the necks of the teeth are very strongly marked.

FIG. 2.

No. 3 is a set of upper teeth made and exhibited at the same time as No. 2.

This cut more especially shows the swell near the necks of the teeth.

FIG. 3.

These cuts show beyond doubt that the imitations of the natural forms of artificial gum, claimed as new in the patent, are not even original copies of nature.

To show where there is originality in this manufacture we quote from the report of the Judges of the Centennial Exposition, 1876.

Referring to the state of the manufacture about the year 1835, they say:

"Then a rubber base was introduced, and from that time the entire dental business has been revolutionized. He (Mr. Justi) made a specialty at that time of supplying manufacturers with moulds of sectional teeth. There were the two modes of manufacturing these teeth, but neither worked to good advantage, one mode being that in which all the colors were put into the mould, and this resulted in bad colors of the gums, owing to the construction of the mould. By the other mode the blue and yellow colors were put into the mould first; then the material was taken out of the mould and carefully put into the first fire to undergo a slight degree of heat, called a 'biscuiting,' and the gum was next applied with a brush, and then fused or burned ready for use. Seeing that in this latter mode there was room for improvement, he commenced to make experiments, and succeeded in constructing moulds suitable to the various formations of the jaws, *adopting curved lines in which he could sink any depth around the neck of the teeth* to receive the gum color, and temporizing the materials so that in one very easy operation he had the teeth ready to finish. The results created general admiration, the teeth being light in bulk, and remarkably life-like in their appearance, the gums especially so. In order to distinguish this make of teeth from others, he adopted a trade-mark accompanied with his name. *This mode of manufacturing artificial teeth has since been copied by all other manufacturers.*"

For these improvements which rendered the present perfection of this manufacture possible, and which, as stated in the report, " were copied by all other manufacturers," Mr. Justi neither received nor asked any patents, but has always been willing that others should profit by following as nearly as they could in his footsteps; his own productions being so far in the advance that imitators caused him neither fear nor jealousy.

In this connection there are a few points worthy of special consideration.

Patents should be granted under such conditions that the patentee will have some assurance that the rights nominally conveyed to him are really guaranteed by the authority of the United States.

Under the present construction of the laws, the patentee is only guaranteed the right to a law suit, and this is so well understood that the intending purchaser of a patent first of all institutes a careful and expensive search to ascertain whether there is any real value in it.

Another abuse of patent privileges is in procuring patents and not manufacturing the thing patented. This dog in the manger style is entirely contrary to the purpose for which patents are granted, that is the public good.

A provision that if, after a reasonable time, the thing presented was not offered for sale the patent should be void, would remedy this evil and permit others to supply the desired article. Valuable improvements are often locked up because manufacturers fear competition with their own productions.

The expediency of patent laws has been a subject of much discussion, but, without entering upon that question, I believe all will agree that there should be radical improvement in their administration.

## Figures and Tables

**Fig. 1. f1:**
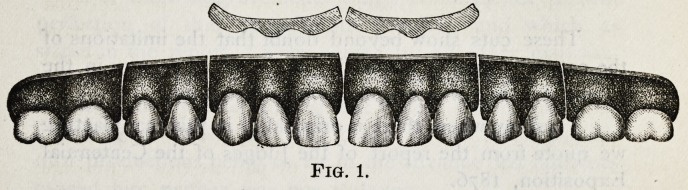


**Fig. 2. f2:**
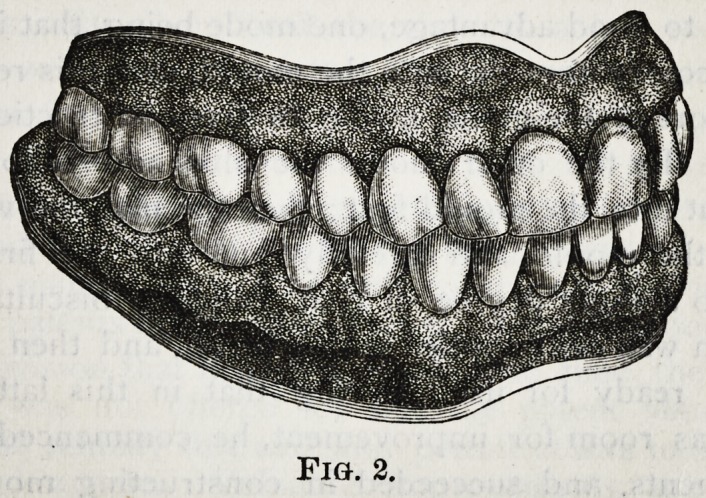


**Fig. 3. f3:**